# Dataset: The Linked Personnel Panel (LPP)

**DOI:** 10.1016/j.dib.2019.104824

**Published:** 2019-11-16

**Authors:** Michael Haylock, Patrick Kampkötter

**Affiliations:** University of Tuebingen, Germany

**Keywords:** Matched employer-employee data set, Management practices, Employer survey, Employee survey

## Abstract

This data in brief article describes the Linked Personnel Panel, a unique longitudinal employer-employee data set, which is representative for German establishments in the private sector with at least 50 employees. The LPP links employee-level information (e.g., about attitudes, preferences and personality) with establishment-level information on management practices and structural firm characteristics.

The LPP up to now contains information on more than 7000 randomly drawn employees aged between 18 and 74 working in 700 to 1200 establishments in three survey waves 2012, 2014 and 2016.

The LPP can be used by any researcher via remote access and/or on-site use.

The LPP is used in the article “The Role of Preferences, Attitudes, and Personality Traits in Labor Market Matching” published in Economics Letters [2].

**Table:** Specifications Table

Subject	Economics and Econometrics
Specific subject area	Personnel Economics; Labor Economics; Human Resource Management; Empirical Evaluation of Human Resource Management Practices
Type of data	Matched employer-employee dataset “Linked Personnel Panel”
How data were acquired	Employee and establishment surveys in the years 2012, 2014, and 2016 (status quo) among German establishments and its workforce
Data format	Access to the raw data set (Stata files) is provided by the Research Data Centre (FDZ) of the German Federal Employment Agency at the Institute for Employment Research (IAB) to any researcher interested in working with the data
Parameters for data collection	Employer survey: First wave (starting) sample was drawn based on the IAB establishment panel wave of 2011. Exempted were establishments with less than 50 employees and establishments from the business sectors of agriculture, forestry and fishery, as well as civil service and charity organisations. The establishments were then randomly drawn from a matrix stratified by sector, establishment size and region. Employee survey: The employee survey is based on a selection of the establishments interviewed. A sample of 7500 employees was to be realised in these establishments. The sampling is based on the Employee History of the IAB (Beschäftigtenhistorik – BeH). The population for drawing the employees comprises 300,881 individuals from 869 establishments, from which a sample of around 38,000 addresses was drawn in October 2012. For survey stratification, the establishments were used according to their size to make sure that a sufficient number of employees could be surveyed for each establishment.
Description of data collection	Employee survey: Computer-aided telephone interviews (CATI) Employer survey: Personal interviews Further information and all questionnaires can be downloaded from the webpage of the Research Data Centre (FDZ) of the German Federal Employment Agency at the Institute for Employment Research (IAB) Link to the webpage: https://fdz.iab.de/en/Integrated_Establishment_and_Individual_Data/lpp/LPP1617.aspx
Data source location	Institution: Research Data Centre (FDZ) of the German Federal Employment Agency at the Institute for Employment Research (IAB) City: Nuremberg Country: Germany
Data accessibility	The data set used in this article, the Linked Personnel Panel, is open to any researcher and is available via the Research Data Centre (FDZ) of the German Federal Employment Agency at the Institute for Employment Research (IAB). Repository name: Research Data Centre (FDZ) of the German Federal Employment Agency at the Institute for Employment Research (IAB) Data identification number: https://doi.org/10.5164/IAB.LPP1617.de.en.v1 Direct URL to data: https://fdz.iab.de/en/Integrated_Establishment_and_Individual_Data/lpp/LPP1617.aspx
Related research article	Author's name: Michael Haylock and Patrick Kampkötter Title: The Role of Preferences, Attitudes, and Personality Traits in Labor Market Matching Journal: Economics Letters DOI: https://doi.org/10.1016/j.econlet.2019.108718 Manuscript number: EL47695 [2]

**Table:** 

**Value of the Data** • The LPP links employer-level information about HR policies with employee-level information about attitudes and behaviour, enabling researchers to analyse how individuals perceive their work and how they respond to HR policies • Researchers in the areas of human resources management, personnel economics, labour economics, and applied psychology can benefit from these data • The longitudinal dimension of the LPP facilitates the analysis of causal effects of HR policies on various outcome measures by eliminating time-constant unobserved variables • A unique feature is the possibility to link both dimensions of the LPP to various external administrative data sets and enrich the available information across a number of dimensions

## 1 Data

The Linked Personnel Panel is a unique longitudinal employer-employee data set, which is representative for German establishments in the private sector with at least 50 employees. The LPP links employee-level information (e.g., about attitudes, preferences and personality) with establishment-level information on management practices and structural firm characteristics. The data set has been developed by a joint research team from the Universities of Cologne and Tuebingen, the Centre for European Economic Research (ZEW) Mannheim and the Institute for Employment Research (IAB) Nuremberg on behalf of the German Federal Ministry of Labor (BMAS).

The LPP up to now contains information on more than 7000 randomly drawn employees aged between 18 and 74 working in 700 to 1200 establishments in three survey waves 2012, 2014 and 2016.

A special feature of the data set is the possibility to link the survey data from the LPP with administrative data (social security records), if respondents have given their informed consent. On the part of the surveyed employees, the data may be merged with their employment biographies since entering the labour market or since 1975, and, on the part of the establishments, with the Establishment History Panel (BHP) entailing all information since the establishment was founded or since 1975.

The administrative data, in particular, are also available for future times when the establishment or the employees no longer take part in the LPP survey. This allows, for example, analyses of medium- and long-term developments such as the period of employment in an establishment.

A detailed description of the data set is provided in Ref. [1] and via the following webpage: https://fdz.iab.de/en/Integrated_Establishment_and_Individual_Data/lpp/LPP1617.aspx.

## 2 Experimental design, materials, and methods

A detailed description of the project background, the data collection method, sampling, weighting, stratification, and numbers of observations is available on the project webpage. Here, we provide methodological reports and a description and codebook of all variables available in the data set. Also, Stata code files for setting up a panel data set as well as test data sets are available on the webpage.

The webpage can be reached via the following URL: https://fdz.iab.de/en/Integrated_Establishment_and_Individual_Data/lpp/LPP1617.aspx.
